# Aluminium-phthalocyanine chloride nanoemulsions for anticancer photodynamic therapy: Development and *in vitro* activity against monolayers and spheroids of human mammary adenocarcinoma MCF-7 cells

**DOI:** 10.1186/s12951-015-0095-3

**Published:** 2015-05-13

**Authors:** Luis Alexandre Muehlmann, Mosar Corrêa Rodrigues, João Paulo Figueiró Longo, Mônica Pereira Garcia, Karen Rapp Py-Daniel, Aline Bessa Veloso, Paulo Eduardo Narciso de Souza, Sebastião William da Silva, Ricardo Bentes Azevedo

**Affiliations:** Faculty of Ceilandia, University of Brasilia, Federal District, Brazil; Department of Genetics and Morphology, Institute of Biological Sciences, University of Brasilia, Federal District, Brazil; Department of Physics, University of Brasilia, Federal District, Brazil

**Keywords:** Spontaneous emulsification, Cancer, Drug delivery systems, Third-generation photosensitizers, Human breast adenocarcinoma MCF-7 cells, Spheroids

## Abstract

**Background:**

Photodynamic therapy (PDT) combines light, molecular oxygen and a photosensitizer to induce oxidative stress in target cells. Certain hydrophobic photosensitizers, such as aluminium-phthalocyanine chloride (AlPc), have significant potential for antitumor PDT applications. However, hydrophobic molecules often require drug-delivery systems, such as nanostructures, to improve their pharmacokinetic properties and to prevent aggregation, which has a quenching effect on the photoemission properties in aqueous media. As a result, this work aims to develop and test the efficacy of an AlPc in the form of a nanoemulsion to enable its use in anticancer PDT.

**Results:**

The nanoemulsion was developed using castor oil and Cremophor ELP®, and a monodisperse population of nanodroplets with a hydrodynamic diameter of approximately 25 nm was obtained. While free AlPc failed to show significant activity against human breast adenocarcinoma MCF-7 cells in an *in vitro* PDT assay, the AlPc in the nanoemulsion showed intense photodynamic activity. Photoactivated AlPc exhibited a 50 % cytotoxicity concentration (CC50) of 6.0 nM when applied to MCF-7 cell monolayers and exerted a powerful cytotoxic effect on MCF-7 cell spheroids.

**Conclusion:**

Through the use of spontaneous emulsification, a stable AlPc nanoemulsion was developed that exhibits strong *in vitro* photodynamic activity on cancer cells.

**Electronic supplementary material:**

The online version of this article (doi:10.1186/s12951-015-0095-3) contains supplementary material, which is available to authorized users.

## Background

Photodynamic therapy (PDT), as a cancer-treatment method, has a series of advantages over approaches such as surgery, chemotherapy and radiotherapy, as discussed elsewhere [1–5]. PDT is based on the production of oxidative species by a photosensitizer (PS), which is a molecule capable of converting specific light energy into chemical potential. In aerobic cells, the photoactivation of PS by a specific, targeted light source converts triplet oxygen (^3^O_2_) into the strongly oxidative species singlet oxygen (^1^O_2_), thereby triggering oxidative stress [2]. The oxidative stress has the potential to induce cell death or a therapeutically significant stress response. Thus, PDT anticancer mechanisms can include direct induction of cancer-cell death [6–8], vascular blockage with subsequent tumour ischemia [9], and increased immune response to tumour antigens [1, 2, 10].

Since the early 1980s, substantial efforts to advance PDT have focused on developing new and improved PS molecules and drug delivery systems [4, 6]. The first generation of PS molecules consisted of complex mixtures of porphyrinoids that were extracted from blood and chemically modified [4, 11]. These molecules exhibited promising anticancer PDT results, but prolonged retention by the skin and other issues hindered clinical application [4]. The second generation of PS molecules showed improved photodynamic activity and chemical purity, and skin accumulation was significantly lower compared to first-generation PS [4, 11]. However, these molecules exhibited low selectivity for tumour cells and were exceedingly hydrophobic [12]. Drug delivery systems, such as polymeric nanoparticles and liposomes, have also been progressing over recent years and currently have the potential to improve upon the second-generation PS and thereby usher in a third generation of PS with new protocols and improved anticancer PDT results [8, 13, 14].

Among second-generation PS, hydrophobic phthalocyanine derivatives are particularly good candidates for being associated to nanostructured drug delivery systems. Hydrophobic phthalocyanines are among the most efficient PS molecules and show excellent accumulation in cancerous cells [12, 15]. On the other hand, hydrophobicity leads to rapid clearance from the body by mononuclear phagocytes and the hydrophobic molecules lose substantial photodynamic activity in aqueous media [12, 16]. Previous works have described nanostructured systems that avoid some hydrophobicity-related drawbacks in aqueous media [6, 7, 9, 14]. Unfortunately, most of these systems employ a high level of organic solvents and are not easily scaled up [6, 17, 18]. This work describes a new nanostructured system containing the hydrophobic PS aluminium-phthalocianine chloride (AlPc), which presents strong anticancer photodynamic activity, and is produced through a simple nanoemulsification method.

## Results and discussion

### Effect of SOR on the nanoemulsion colloidal characteristics

Hydrophobic phthalocyanine derivatives present intense and prolonged accumulation in cancerous cells [12]; however, at the same time, their hydrophobicity is a major drawback. This property confers a low circulation time in the bloodstream because hydrophobic phthalocyanine derivatives are more readily cleared by mononuclear phagocytes [12, 19]. Moreover, hydrophobic photosensitizers aggregate in aqueous systems, such as the cytosol and bloodstream, leading to an intense loss in photodynamic activity due to the quenching effect [20, 21].

In the present work, a nanoemulsion produced by spontaneous nanoemulsification is proposed to improve the photodynamic activity of AlPc. Nanoemulsions are kinetically stable mixtures of immiscible liquids, consisting of a continuous phase containing dispersed nanodroplets (the dispersed phase, generally 20 to 300 nm in diameter) [22, 23], which can be easily produced on a large scale without the need for complex equipment or processes. The method used in this work, spontaneous nanoemulsification, is based on the generation of nanodroplets by the turbulent movement of surfactants from the oil to the oil–water interface after addition of an aqueous phase to the surfactant-oil mixture [22].

**Fig. 1:**
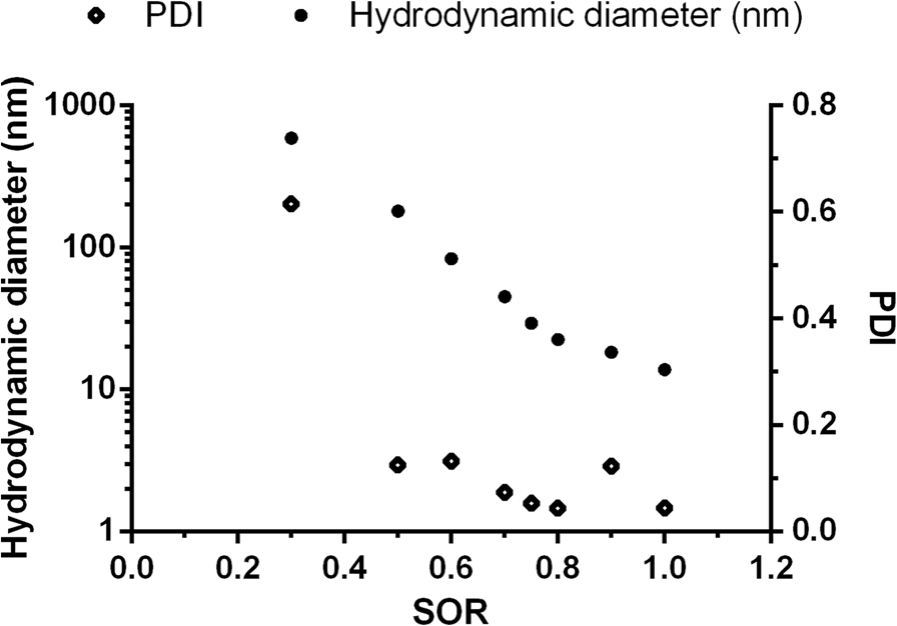
Effect of the surfactant-to-oil ratio (SOR) on hydrodynamic diameter and polydispersity index (PDI) of droplets of emulsions produced by spontaneous emulsification

Cremophor ELP® was used as the surfactant, while castor oil composed the oily phase. All of the nanoemulsions were of the oil-in-water type, as observed in conductivity experiments (data not shown). The average hydrodynamic diameter (HD) of nanodroplets was directly proportional to the surfactant-to-oil ratio (SOR, Fig. 1). This phenomenon has been previously reported [22] and is due to the larger mean volume of oil per droplet. Nanoemulsions with narrow size distributions (polydispersity index – PDI – of approximately 0.1) were obtained with an SOR between 0.5 and 0.9. Anton and Vandamme [22] described nanoemulsions obtained by spontaneous emulsification with Cremophor ELP® as the surfactant and Labrafil M1944 CS® as the oil with a SOR of 0.2. The higher SOR needed for producing castor oil nanoemulsions observed in the present work is probably due to the different chemical composition of castor oil as it is a mixture of triacylglycerols containing hydroxy fatty acids, mainly ricinoleic acid, while Labrafil M1944 CS® is more easily emulsified because it contains polyoxyl moieties and is thus less hydrophobic than castor oil. In subsequent experiments, the SOR was kept at 0.75. This formulation was chosen because of its monodispersion and low average HD of the nanodroplets, which can facilitate the diffusion of nanodroplets in biological tissues [24, 25].

### Effect of the AlPc concentration in the surfactant/oil mixture (SOmix) on the photo-triggered production of reactive species and the colloidal and photophysical properties of nanoemulsions

As the concentration of PS profoundly affects its photodynamic activity [6, 26, 27], nanoemulsions produced with different initial AlPc concentrations were tested for their colloidal, photophysical and photochemical properties.

**Fig. 2:**
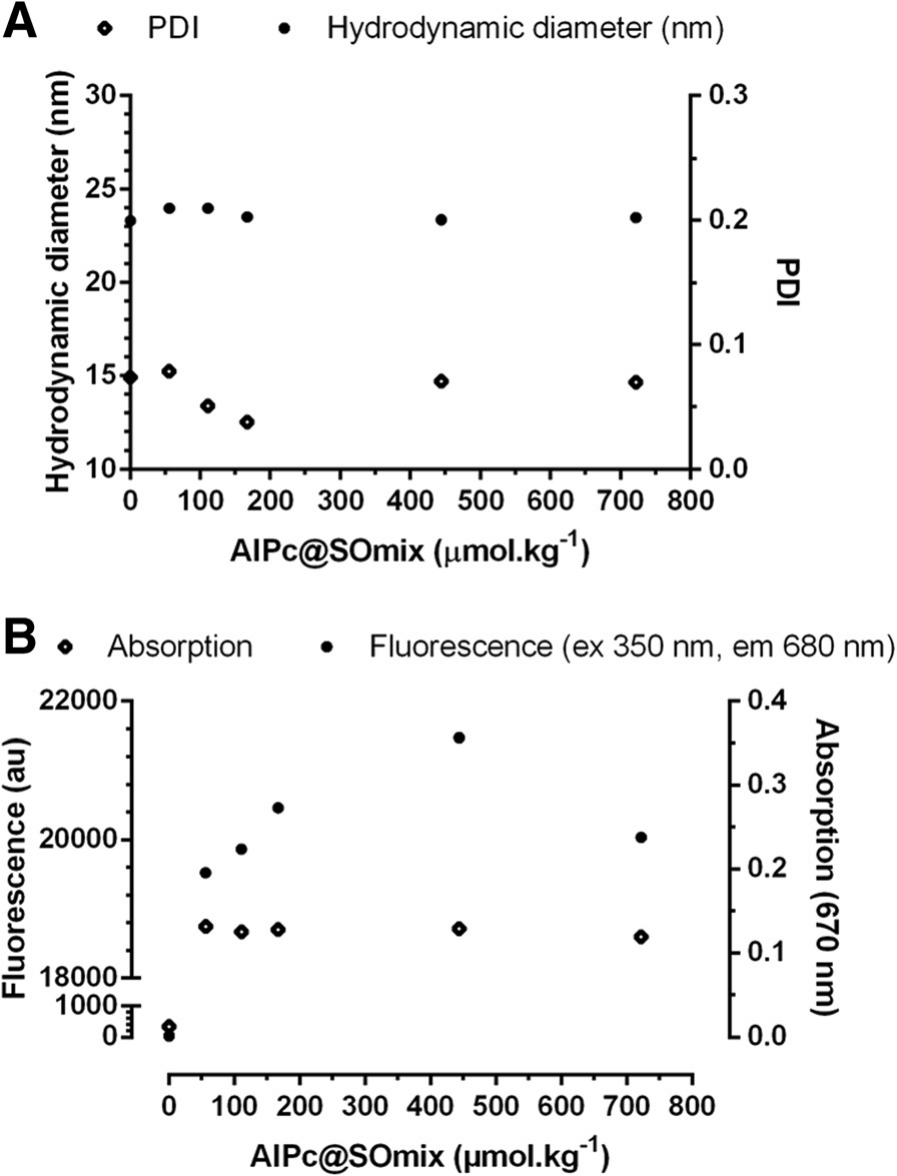
Effects of the AlPc concentration in the surfactant/oil mixture (AlPc@SOmix) on the **(a)** hydrodynamic diameter and polydispersity index (PDI) of nanodroplets and on **(b)** fluorescence (excitation 350 nm, emission 680 nm) and light absorption (670 nm) of nanoemulsions produced by spontaneous emulsification

The concentration of AlPc in the surfactant/oil mixture (SOmix) did not affect the HD and PDI nor did it affect the light absorption of nanoemulsions prepared with a SOR of 0.75 (Fig. 2). However, there was a significant influence of the concentration of AlPc in the SOmix on the fluorescence of nanoemulsions (R_Pearson_ = 1.0 for concentrations ranging from 46 to 444 μmol.kg^−1^, p < 0,05). All of the nanoemulsions, except S0, were diluted to 1.0 μM AlPc prior to fluorescence measurements to ensure that the aggregation state of AlPc was the only factor affecting fluorescence and light absorption.

The system with the highest concentration of AlPc in SOmix tested, S722 (722 μmol.kg^−1^), presented a low fluorescence intensity and low ability to produce reactive oxygen species (ROS) under irradiation. This fact may be explained by the quenching effect. At certain high concentrations of AlPc, similar to the case for other fluorescent molecules, the distance between molecules is short enough to facilitate the quenching effect [6, 26, 27]. The nanoemulsion produced with AlPc 444 μmol.kg^−1^ in SOmix (S444) presented light absorption and fluorescence intensity comparable to that of AlPc in ethanol, which is a good solvent for this molecule. This formulation was then chosen for subsequent experiments.

**Fig. 3:**
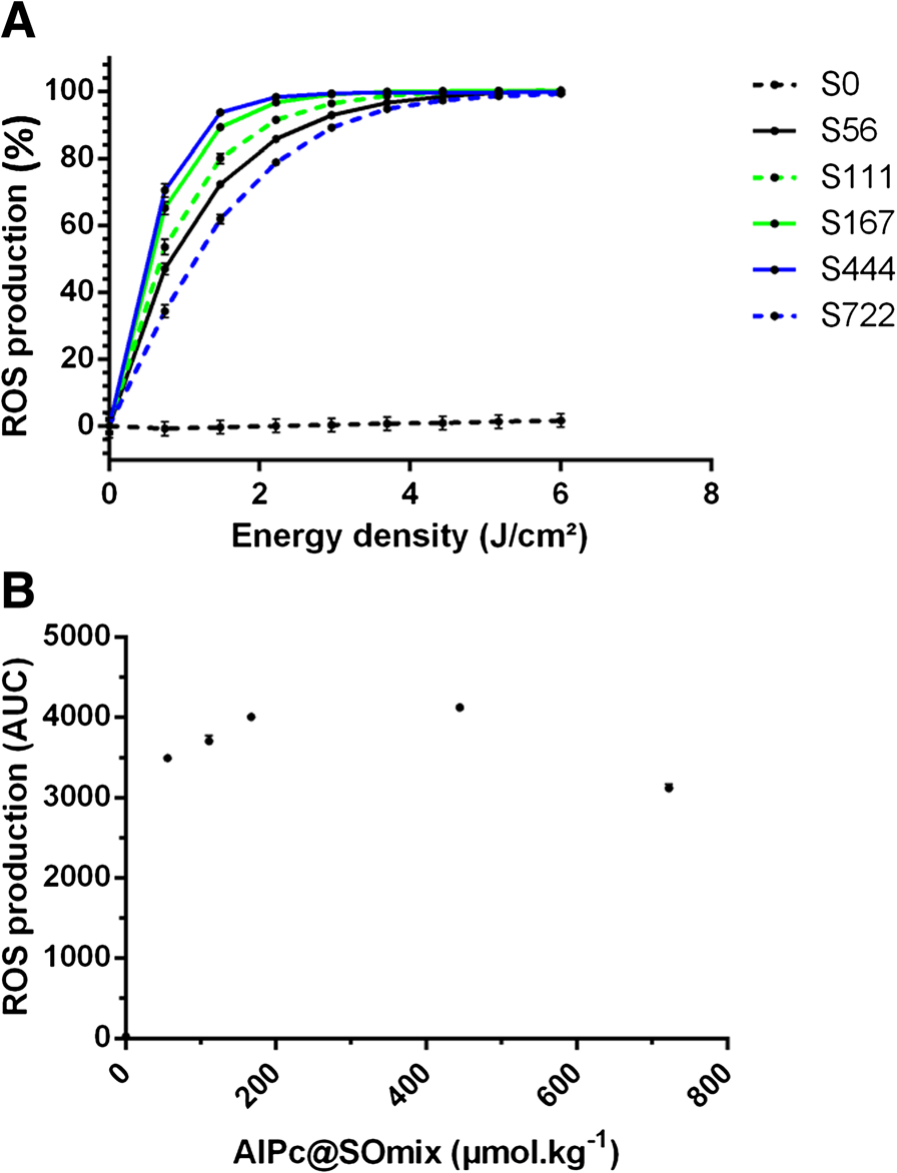
Production of reactive oxygen species (ROS) by nanoemulsions prepared with different concentrations of AlPc (0 to 722 μmol.kg^−1^) in the surfactant/oil mixture (SOmix) with the same final AlPc concentration (1 μM). In **(a)**, the ROS production results are presented as a function of the energy density. In **(b)**, the results are expressed as the area under the curve (AUC) of the values presented in graph A

As expected, the results of photo-triggered production of ROS by nanoemulsions (Fig. 3) match the fluorescence intensity results. The most intense production of ROS was observed for the S167 and S444 nanoemulsions, with their maximum production reached at an energy density of 2.95 J/cm^2^. The lowest rate of ROS generation was obtained with S722, with a maximum production reached only at 5.18 J/cm^2^. As expected, S0 did not produce ROS under irradiation.

The colloidal properties of S444 and S0 in different dispersants are presented in Table 1. The zeta potential of these formulations was close to zero, reflecting the neutral nature of the nanodroplets, which was expected due to the interfacial stabilization performed by the neutral, hydrophilic polyoxyl moieties of Cremophor ELP® and not by surface charge.

**Table 1:** Colloidal properties of S444 and S0 nanoemulsions in different dispersants

Nanoemulsion/Dispersant	Hydrodynamic diameter (nm)	PDI	Zeta potential (mV)
S444/PBS	25.08 ± 0.28	0.131 ± 0.02	- 6.24 ± 0.47
S444/DMEN	25.57 ± 2.34	0.130 ± 0.09	- 3.15 ± 1.54
S444/DMEN F12	25.89 ± 1.23	0.109 ± 0.03	- 2.95 ± 0.96
S0/PBS	24.33 ± 0.27	0.094 ± 0.03	- 3.84 ± 0.56

**Fig. 4:**
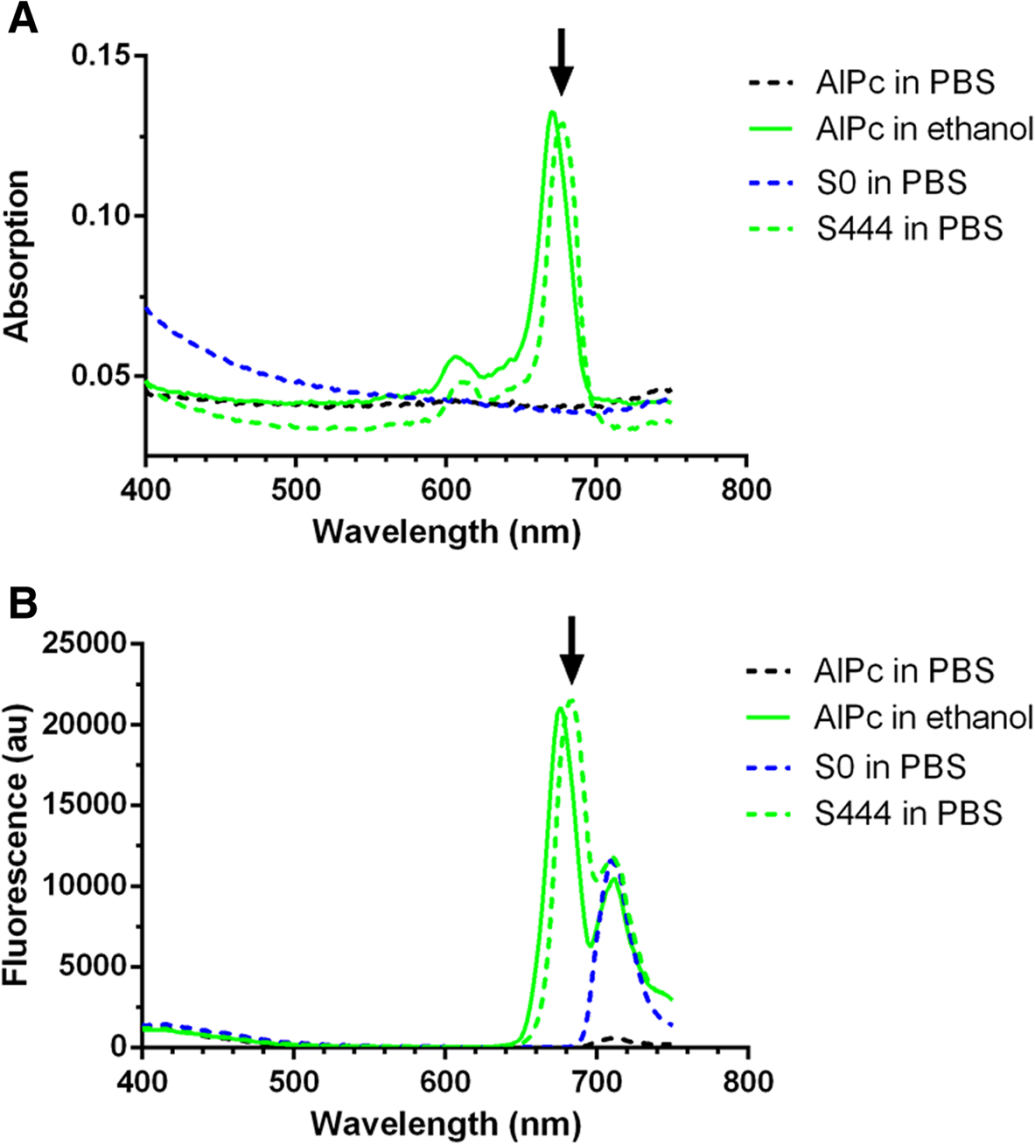
Absorption **(a)** and fluorescence (**b**, excitation at 350 nm) spectra of 1 μM free AlPc in PBS or ethanol and of a blank nanoemulsion (S0) and S444 (1 μM AlPc) dispersed in PBS. The peaks at 710 nm in **b** are artefacts of the equipment

The absorption and fluorescence spectra of S444 and S0 are presented in Fig. 4. As expected, free AlPc neither significantly absorbed light nor emitted fluorescence when dispersed in water. However, when dissolved in ethanol, AlPc absorbs light with a peak at 670 nm and emits fluorescence at 674 nm when excited with light at 350 nm. AlPc in the S444 nanoemulsion absorbed light with a peak at 674 nm and emitted fluorescence at 684 nm when excited with light at 350 nm. Both the absorption and fluorescence intensity of AlPc in S444 were close to those observed in ethanol. As expected, S0 neither presented significant absorption nor fluorescence superposing the peaks presented by AlPc. The peak observed at 710 nm in the fluorescence spectra is an artefact of the equipment.

### Colloidal and photophysical stability of an AlPc nanoemulsion (S444)

The HD of S444 did not change over the evaluation time at the storage temperatures tested (data not shown). Moreover, PDI remained near 0.1 along the period of analysis, reflecting the stability and monodispersion of the nanodroplet size. The AlPc content did not vary significantly during the time of analysis. Additionally, both the absorption of light at 676 nm and fluorescence intensity (excitation 350 nm, emission 680 nm) did not significantly vary over the time of evaluation.

### SERS and Raman spectra

**Fig. 5:**
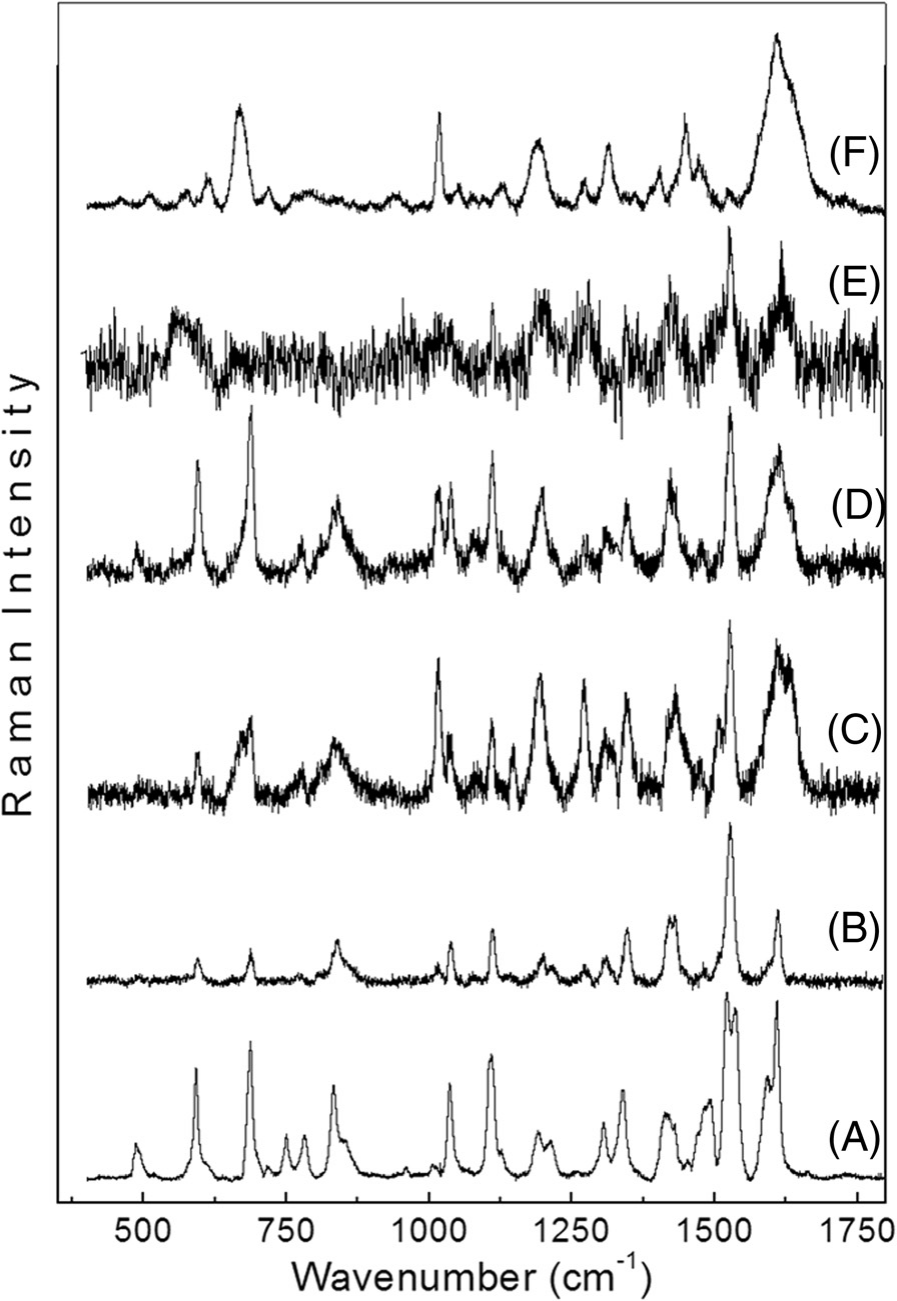
Raman spectrum of pure AlPc powder **(a)** and the SERS spectra of 1 μM AlPc in S56 **(b)**, S167 **(c)**, S444 **(d)**, S722 **(e)**, and 1 μM free AlPc in water **(f)**

In order to verify the aggregation state of AlPc, SERS and Raman spectra of AlPc under different conditions were produced. Fig. 5 presents the SERS spectra of nanoemulsions S56, S167, S444 and S722 (Fig. 5b-e), all of which at 1 μM AlPc. For comparison, the normal Raman spectrum for the AlPc powder (Fig. 5a) and the SERS spectrum of a 1 μM dispersion of free AlPc in water (Fig. 5f) are also shown.

Figure 5a presents a typical Raman spectrum of AlPc powder. Despite the fact that the AlPc molecule displays highly coupled vibrational modes, it is possible to affirm that the Raman spectrum is dominated by vibrations from benzene (at approximately 690, 1110, 1190, 1310, 1340, 1410, 1590 and 1615 cm^−1^), macrocycle (at approximately 590, 750, 830 and 1520 and 1538 cm^−1^) and pyrrole (at approximately 830, 1040 and 1210 cm^−1^) [28]. On the other hand, the SERS spectrum of free AlPc in water is significantly different from the Raman spectrum of AlPc powder. The most remarkable differences are the near complete absence of the vibrational modes from the central region of the AlPc (macrocycle and pyrrole) and the shift in the vibrational modes from the benzene ring.

The surface-enhanced Raman spectroscopy (SERS) spectra of the nanoemulsion samples exhibit similar characteristics to the Raman spectrum of AlPc powder. However, a careful analysis shows important differences. The spectrum for the AlPc powder differs from the spectra of the nanoemulsion samples in relation to the presence or absence of certain bands and in the relative intensities of some peaks. For example, the peak at 750 cm^−1^, assigned to the vibrations of the C_α_-N_α_-C_α_ and Al-N_α_ bonds, is present in the spectrum of the AlPc powder, but the peak is absent in the nanoemulsion spectra. In addition, the decrease in the relative intensities between the vibrations related to the central regions of the AlPc molecules (*I*_*c*_) (peaks at 830, 1040 and 1520 cm^−1^) and the vibrations related to the benzene group located at the edges of the AlPc molecule (*I*_*ed*_) (peaks at 1190, 1410 and 1615 cm^−1^).

These findings suggest an aggregation tendency with increasing concentrations of AlPc in SOmix. This result, particularly, is aligned to the photophysical (fluorescence and light absorption intensities) and photochemical (phototriggered ROS production) observations presented before. It is clear that *I*_*c*_*/I*_*ed*_ decreases with increasing concentrations of AlPc, tending towards the SERS spectrum of AlPc in water, in which the vibrational modes of the central region of the AlPc molecule are absent. For example, the ratio *I*_*c*_*/I*_*ed*_, between the peaks at 1520 and 1410 cm^−1^ is 2.2, 2.0, 1.5, 1.2 and 0.7 for the AlPc powder and the nanoemulsions S56, S176, S444 and S722, respectively. Similar behaviours are observed for the ratios between the peaks at 1036 and 1190 cm^−1^, 1520 and 1615 cm^−1^, and 830 and 1190 cm^−1^.

Aggregation occurs due to AlPc-AlPc interactions involving the large planar aromatic ring systems, and the resulting formation of ordered structures with the AlPc benzene rings was preferentially located on the surface of aggregates [29]. Because the SERS enhancement factor decays with *1/r*^12^, the SERS spectra of aggregates depend on the dominant chemical species closer to Ag nanoparticles used in this method, which are preferentially the benzene rings located on the edge of the AlPc molecule in this case. This finding explains the absence of the modes from the central region of the AlPc molecule and the prevalence of vibrational modes of benzene rings in the SERS spectra.

### S444-mediated PDT in monolayers of MCF-7 and MCF-10A cells *in vitro*

As nanoemulsion S444 showed better photodynamic activity due to low AlPc aggregation, this formulation was used for the *in vitro* tests with cells. At all the concentrations tested, S444 did not significantly affect the viability of cancerous (MCF-7) and non-cancerous (MCF-10A) cells in the dark. Absence of toxicity in the dark is a general requirement for PS systems [4, 30, 31] because it assures that only the tissues containing the PS and that are irradiated will be affected by the toxic effects of PDT. LED light alone did not significantly affect the viability of tested cells (data not shown). Moreover, S0 (blank nanoemulsion) was not cytotoxic to MCF-7 or MCF-10A cells in the dark or after irradiation at concentrations equivalent to those tested for S444 (see Additional file 1: Figure S1 in supplementary information).

**Fig. 6:**
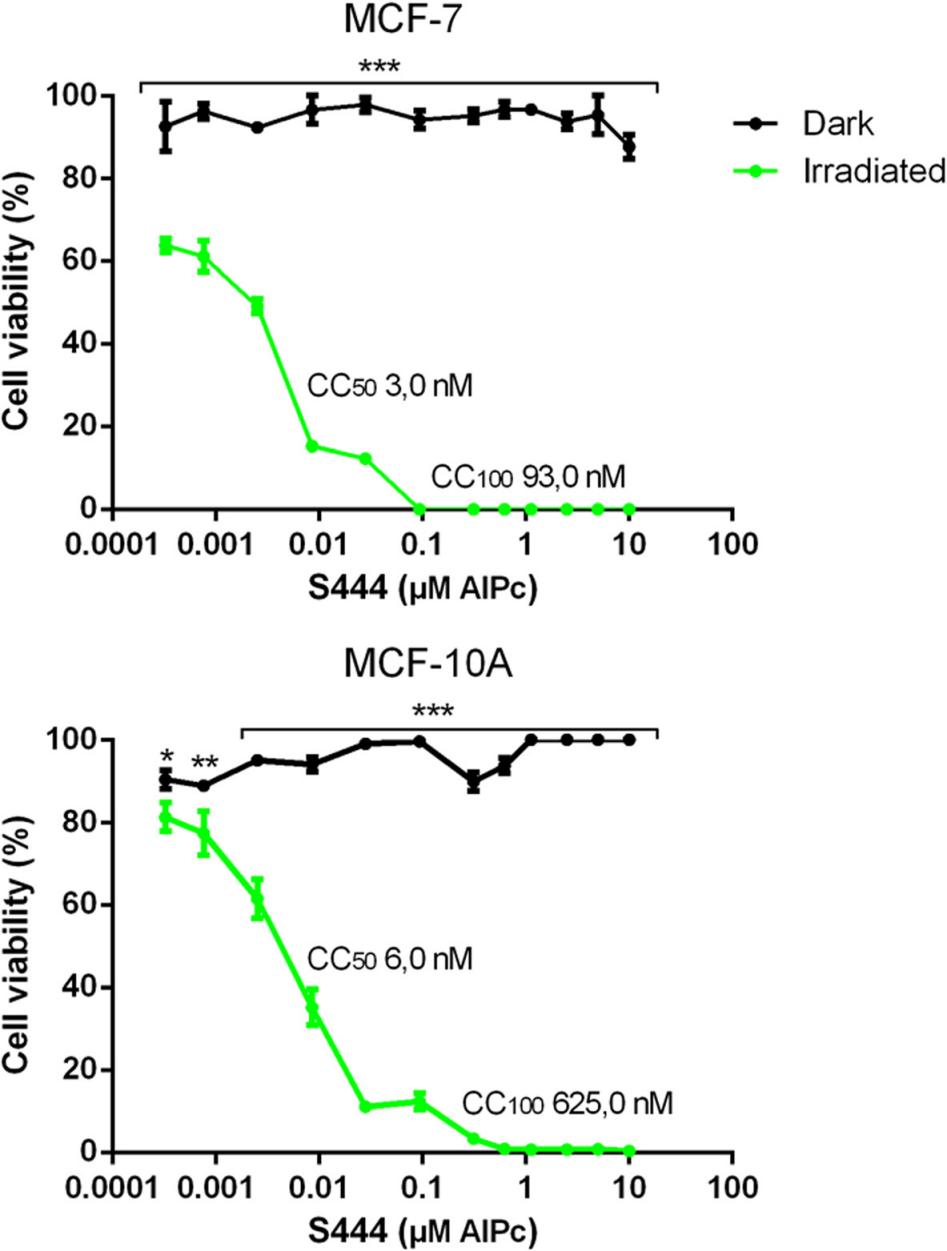
Viability of cancerous (MCF-7, human) and non-cancerous (MCF-10A, human) cells exposed to different concentrations of S444 for 15 min, and then kept in the dark or irradiated (660 nm, 4.4 J/cm^2^). Cell viability was evaluated by the MTT method 24 h after treatment. The CC_50_ and CC_100_ of S444 after irradiation were 3.0 and 93.0 nM, respectively. *p < 0.05, **p < 0.01, and ***p < 0.001, for dark vs irradiated

MCF-7 and MCF-10A cells showed different susceptibilities to S444 after irradiation with LED light (660 nm, 4.4 J/cm^2^). As shown in Fig. 6, there was a significant decrease in MCF-7 cell viability in all of the S444 AlPc concentrations tested (p < 0.001), with 50 % cytotoxic concentration (CC_50_) and 100 % cytotoxic concentration (CC_100_) of 3.0 and 93.0 nM, respectively. For MCF-10A, PDT reduced cell viability, although these cells were less affected in comparison to MCF-7 cells, with photoactivated S444 presenting a CC_50_ and CC_100_ of 6.0 and 625.0 nM, respectively. Thus, the values of CC_50_ and CC_100_ of S444 in MCF-10A for photoactivated S444 were 2- and 6.7-fold higher, respectively, compared to those observed in MCF-7 cells.

The more intense effect against MCF-7 cells may be due to the higher endocytic activity that is frequently observed in cancerous cells [32]. A similar result was observed in a previous work involving AlPc in polymeric nanoparticles, which were more active against MCF-7 cells in comparison to MCF-10A cells [6]. That work also showed that MCF-7 cells were more sensitive to PDT mediated by AlPc in nanoparticles.

### S444-mediated PDT against spheroids of MCF-7 cells *in vitro*

**Fig. 7:**
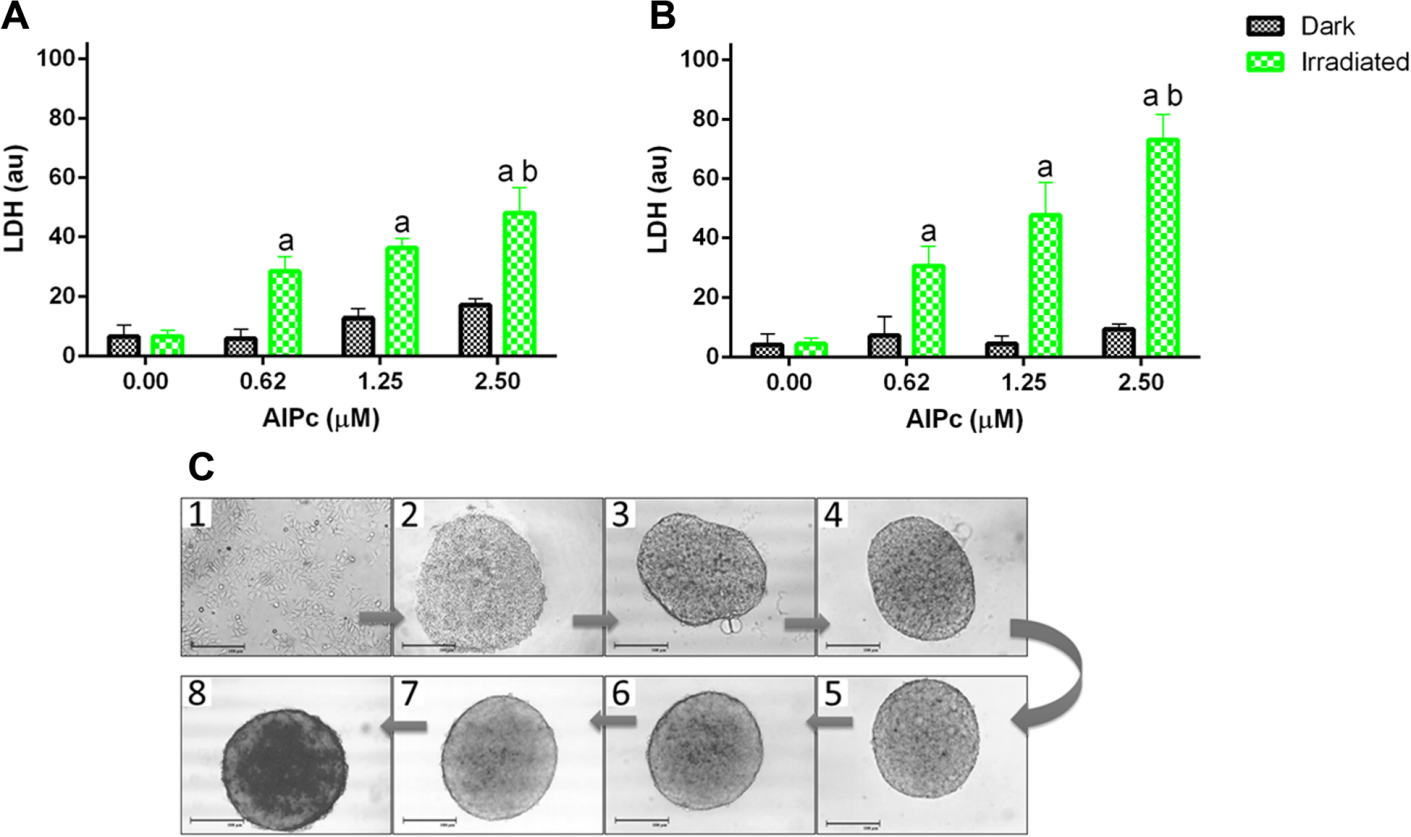
Viability of cancerous cell (MCF-7, human) spheroids expressed as arbitrary units (au) of lactate dehydrogenase (LDH) activity in the culture supernatant (**a** and **b**). Spheroids were exposed to different concentrations of S444 (expressed as μM of AlPc) for 30 min **(a)** or 60 min **(b)** and were then kept in the dark or irradiated (660 nm, 4.4 J/cm^2^). The formation of a spheroid is shown in **c** (scale bar, 100 μm), including: 1) a MCF-7 monolayer, 2) MCF-7 cells after centrifugation, and 3 to 8) the evolution of the spheroid from the 1^st^ to 6^th^ day. ^a^p < 0.001 vs dark conditions at the same AlPc concentration, and vs 0.00 μM AlPc irradiated. ^b^p < 0.01 vs 0.62 and 1.25 μM irradiated

Figure 7c shows the evolution of MCF-7 cells in culture from a monolayer to a spheroid of approximately 200 μm in diameter. In comparison to cell monolayers, *in vitro* cell spheroids more accurately mimic the architecture of tumours, which show tumour cells on the surface while hiding other cells on spheroids [33, 34]. MCF-7 cell spheroids were sensitive to S444-mediated PDT *in vitro*. As expected, significant toxicity to MCF-7 cells was observed only when S444 and light irradiation were combined. This effect was dependent on the concentration of AlPc. Irradiation or S444 alone did not significantly induce spheroid cells lysis.

The concentration- and exposure time-dependent pattern of toxicity induction is probably associated with the extension of AlPc permeation in spheroids. When spheroids were exposed for 30 min to S444 at 1.25 and 2.50 μM AlPc, PDT increased the release of LDH by only 2.8-fold. However, when the exposure time was increased to 60 min, PDT based on S444 at 1.25 and 2.50 μM AlPc induced, respectively, 10.5- and 7.8-fold increases in LDH release. Thus, S444-mediated PDT induced an intense lysis of cancer cell spheroids to an even greater extent than that reported in previous works with conventional chemotherapeutic drugs [34]. This result shows that S444 has a great potential for permeating cancer cell spheroids, since its efficacy is directly dependent on permeation through spheroids. Therefore, future *in vivo* anticancer tests with S444 must take into account the permeation time of S444 in tumour tissues to optimize the PDT protocol.

### Cellular distribution of S444 in MCF-7 cells

**Fig. 8:**
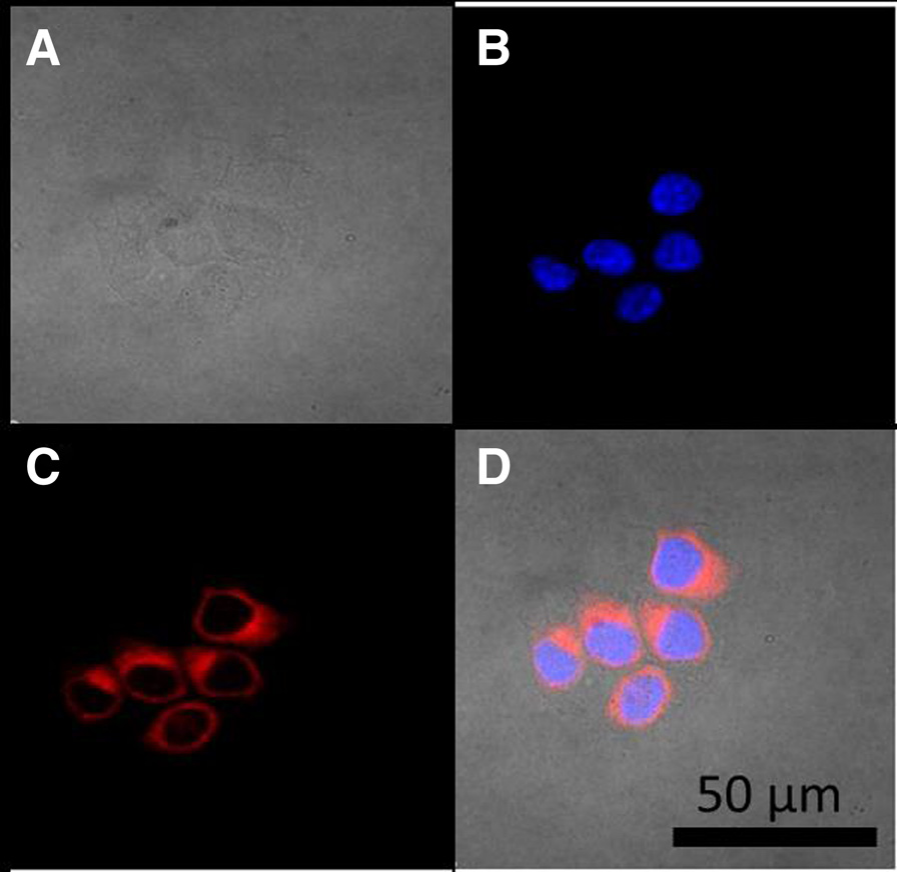
Internalization of S444 (125 nM AlPc) by MCF-7 cells *in vitro* after 15 min of exposure. AlPc appears in red, while nuclei are stained blue by 4',6-diamidino-2-phenylindole (DAPI). **a)** bright field. **b)** cell nuclei in blue. **c)** AlPc in red. **d)** merge of **a**, **b** and **c**

MCF-7 cells exposed for 15 min to S444 presented AlPc in the cytoplasm but not in nuclei, as shown in Fig. 8. The oxidative species generated by a photoactivated photosensitizer diffuse for only a few nanometers [2], being thus restricted to the site where the photosensitizer is concentrated. Thus, the AlPc distribution pattern observed in this study may prevent PDT-mediated DNA damage and resistance-inducing modifications in the cell genome [1].

## Conclusion

This work reported the development of a nanoemulsion containing AlPc, a hydrophobic phthalocyanine derivative. This formulation showed intense photodynamic activity in aqueous media and thus was effective in reducing the viability of adenocarcinoma MCF-7 cells *in vitro*, both as monolayers and spheroids. It remains to be tested in *in vivo* tumour models, however, whether further selectivity for tumours may be achieved with the use of this nanoemulsion.

## Materials and methods

The reagents employed in this work are as follows: Dulbecco’s modified Eagle’s medium (DMEM, HyClone, Logan, USA); DMEM:F12 (HyClone, Logan, USA); Penicillin, streptomycin and foetal bovine serum (Gibco, Grand Island, NY, USA); Anti-fading agent (ProLong Gold, Life sciences); Phosphate-buffered saline (Laborclin, Pinhais, Paraná, Brazil); Dimethyl sulfoxide and ethanol (Vetec, Xerém, Rio de Janeiro, Brazil); HPLC-grade methanol and ethanol (Mallinckrodt Inc., Hazelwood, MO, USA); ACS-grade trifluoroacetic acid (TFA) (Vetec, Duque de Caxias, RJ, Brazil); Milli-Q water (Barnstead EASYpure II Thermo Scientific, San Jose, CA, USA); Cell lines: human mammary adenocarcinoma MCF-7 cells (Rio de Janeiro Cell Bank [RJCB], Rio de Janeiro, Brazil), human mammary epithelial MCF-10A cells (kindly provided by Dr. Maria Mitzi Brentani, University of São Paulo, São Paulo, Brazil). All other materials were purchased from Sigma (São Paulo, Brazil).

### Production of nanoemulsions with different surfactant-to-oil ratios

Cremophor ELP® (polyoxyl–35 castor oil) was used as a surfactant [22]. Different mixtures of Cremophor ELP® and castor oil (SOmix) were produced for final surfactant-to-oil weight ratios (SOR, SOR = surfactant weight/surfactant weight + oil weight) ranging from 0.3 to 1.0. Then, 70 mL of distilled water were added to 12 g of each SOmix at RT with mild magnetic stirring (300 RPM for 15 min). Next, the volume was adjusted to 100 mL with distilled water, and the systems were kept at RT for 24 h. The hydrodynamic diameter (HD, Z-average size), zeta potential, and PDI of droplets dispersed in each system were then measured according to the protocol in the section titled “Colloidal properties”.

### Production of nanoemulsions with different concentrations of AlPc in SOmix

Nanoemulsions were prepared with a fixed amount of SOmix (75 % Cremophor ELP® and 25 % castor oil, w:w, SOR = 0.75) containing different concentrations of AlPc. In this experiment, AlPc was first dissolved in ethanol 99 °GL at a concentration of 1.7 mM. Then, different volumes of the AlPc solution were added to 12 g of SOmix aliquots so that the AlPc concentrations ranged from 56 to 722 μmol.kg^−1^. Ethanol was then removed at 100 °C under mild magnetic stirring (300 RPM for 15 min). Next, solutions of AlPc in SOmix were left to cool to RT, and 70 mL of distilled water were added under mild stirring. These systems were further stirred for 15 min at RT, and the volumes were adjusted to 100 mL with distilled water. A nanoemulsion without AlPc was prepared through this same procedure (S0). Throughout this work, nanoemulsions are named by their respective AlPc concentrations in the SOmix preceded by the letter S, e.g., “S56” represents the system prepared with SOmix containing AlPc at 56 μmol.kg^−1^. This nomenclature was adopted to distinguish the concentration of AlPc in the SOmix from the concentration of AlPc in the final nanoemulsion. Nanoemulsion properties were analysed according to the sections titled “Colloidal properties”, “Photophysical properties” and “Detection of reactive oxygen species”.

### Colloidal properties

The hydrodynamic diameter (HD) and zeta potential of droplets dispersed in water were measured at 25 °C by photon correlation spectroscopy and electrophoretic laser Doppler velocimetry (ZetaSizer Nano ZS®, Malvern Instruments, Malvern, UK), respectively. The polydispersity index (PDI) was calculated using the equipment software for DLS measurements. All measurements were performed in triplicate and the results are presented as the mean ± SEM.

### Photophysical properties

The fluorescence intensity and light absorption were measured with a spectrophotometer (Spectramax® M2, Molecular Devices, Sunnyvale, CA, USA) at 25 °C in 96-well microplates. In all experiments, nanoemulsions were diluted to 1 μM AlPc before fluorescence or absorption measurements were performed.

### Surface-enhanced raman spectroscopy (SERS)

Silver colloid was prepared by reducing AgNO_3_ with an excess of trisodium citrate, Na_3_C_6_H_5_O_7_ [35]. Then, 25 μL of each nanoemulsion sample was diluted in aliquots of 25 μL of silver colloid, and Raman spectra were immediately recorded. The Raman (for pure AlPc powder) and SERS spectra (for all other samples) were obtained in the backscattering configuration and analysed using a triple spectrometer (Jobin Yvon Model T64000; Horiba, Tokyo Japan) equipped with a CCD detector. Samples were illuminated at 488 nm with an Argon ion laser at an optical power of 20 mW.

### AlPc quantification by HPLC

The chromatographic equipment (Shimadzu-Prominence) consisted of an on-line degasser (Model DGU 20A5), solvent delivery module (Model LC-20AT), autosampler (Model SIL-20AHT), column oven (Model CTO-20A), fluorescence detector (Model RF-10AXL) and system controller CBM-20A. A reverse-phase C8 column Vydac of 5 μm, 4.6 mm × 250 mm (Thermo Fischer Scientific, Massachusetts, USA) with a pre-column of 5 μm, 4.6 mm × 50 mm (Thermo Fischer Scientific, Massachusetts, USA) was used. The mobile phase consisted of a mixture of 0.12 % (m:v) TFA in Milli-Q water (pump A) and methanol (pump B) at 40:60 (v/v) rendering an isocratic phase. Fluorimetric measurements were carried out in a 12-μL flow cell at 610 and 675 nm excitation and emission wavelengths, respectively. The injection volume was 5 μl and the flow rate was 1 ml/min at a working pressure of 135 kgf.cm^−2^. Analyses were performed with a column temperature of 30 °C. LCsolution Software (Shimadzu, Tokyo, Japan) was used for data processing.

The calibration curve was generated with AlPc solutions with concentrations ranging from 0.01 to 8 μM. For AlPc quantification in nanoemulsions, sample aliquots were dissolved in ethanol (1:40, v/v), vortexed for 3 min, filtered through 0.22-μm nylon filters (Millex GN, Millipore, Darmstadt, Germany), and injected into the HPLC system.

### Nanoemulsion stability

The colloidal and photophysical stability of S444 was evaluated for 365 days. Briefly, 200-μL aliquots of S444 were kept in the dark at 4 °C, 25 °C or 37 °C. At specific times of storage, one aliquot of each nanoemulsion was tested for nanodroplet hydrodynamic diameter, PDI (see section “Colloidal properties”), light absorption (676 nm), fluorescence intensity (excitation at 350 nm, emission at 680 nm) (see section “Photophysical properties”), and AlPc content (see section “AlPc quantification by HPLC”).

### Detection of reactive oxygen species

Reactive oxygen species (ROS) were detected by an indirect method, employing the probe 1,3-diphenylisobenzofuran (DPBF), as described elsewhere [36, 37]. In a typical experiment, 10 μL of DPBF in ethanol (225 μg/mL) was added to 200 μL of sample in a transparent 96-well microplate. Then, absorption at 410 nm was recorded at 25 °C with a spectrophotometer before and immediately after irradiation of the sample with light energy densities ranging from 0.1 to 6.0 J/cm^2^. The absorption values were normalized, and the optical density at 410 nm before irradiation was considered to be 0 %, while the lower plateau absorption values were considered to be 100 % ROS generation. The results were expressed as ROS production (%) as a function of energy density or area under the curve (AUC). This experiment was performed in triplicate.

### Cell culture

MCF-7 cells were cultured in DMEM, supplemented with 10 % (v:v) foetal bovine serum and a 1 % (v:v) antibiotic solution (100 IU/mL penicillin and 100 mg/mL streptomycin). MCF-10A cells were cultured in DMEM:F12 (1/1, v:v) supplemented with 5 % (v:v) equine serum, 20 ng/mL epidermal growth factor (EGF), 10 μg/mL bovine insulin, 0.5 μg/mL hydrocortisone, 100 ng/mL cholera toxin, and 1 % (v:v) antibiotic solution (100 IU/mL penicillin and 100 mg/mL streptomycin). All cells were maintained at 37 °C in a 5 % CO_2_ humidified atmosphere.

### Intracellular distribution of S444

The intracellular localization of the AlPc nanoemulsion S444 was visualized by confocal microscopy. Briefly, MCF-7 cells were cultured on coverslips placed in 24-well plates for 24 h according to the conditions described in the “Cell culture” section at a density of 2 × 10^4^ cells/well. Next, the cells were exposed to S444 diluted in culture medium at a concentration equivalent to 125 nM AlPc for 15 min at 37 °C, 5 % CO_2_ and humidified atmosphere. Then, the cells were washed twice with PBS, fixed with 4 % (w:v) paraformaldehyde for 15 min, stained with DAPI, washed twice with PBS, and mounted on glass slides with anti-fading agent. Next, the cells were visualized by a confocal microscope (Leica, TCS SP5, São Paulo, Brazil). AlPc fluorescence was detected with excitation at 405 nm and emission at 633 nm. DAPI fluorescence was detected with an excitation at 358 nm and emission at 461 nm.

### Formation of MCF-7 cell spheroids

Spheroids were obtained based on a protocol described elsewhere [34]. Briefly, 50 μL of 1.5 % low melting agarose (w:v in distilled water) were added to the wells of a 96-well microplate. Next, 1.5 × 10^4^ MCF-7 cells, which were processed as described in the “Cell culture” section, were seeded on each well, and the microplate was then centrifuged for 15 min at 1500 × *g*. Microplates were maintained at 37 °C in a 5 % CO_2_ humidified atmosphere. Formation of spheroids was monitored daily and photographed with an inverted microscope (Olympus Co., Tokyo, Japan) equipped with a digital camera (Moticam 2300 3.0MP, Life Resolution, Brazil). Microplates were maintained under these culture conditions until spheroids of approximately 200 μm in diameter were obtained (generally, for approximately 6 days). Each well contained one single spheroid. Then, specific treatments were applied according to the “Cell treatment design” section.

### Cell treatment design

MCF-7 and MCF-10A cells in monolayers were maintained as described in the “Cell culture” section and the cells were 1) exposed only to culture medium without additional treatment; 2) irradiated with LED light (660 nm) at an energy density of 4.4 J/cm^2^; 3) exposed to nanoemulsions without AlPc (S0) dispersed in culture medium for 15 min in the dark at castor oil concentrations of 0.19, 0.38 and 0.76 % (w:v), equivalent to 2.5, 5.0 and 10.0 μM AlPc, if compared to S444; 4) exposed to S444 diluted in culture medium for 15 min in the dark at concentrations ranging from 0.3 nM to 10.0 μM AlPc; or 5) exposed to S444 diluted in culture medium for 15 min in the dark at concentrations ranging from 0.3 nM to 10.0 μM AlPc, washed twice with PBS, and then irradiated with LED light (660 nm) at an energy density of 4.4 J/cm^2^. Next, the cells were cultured for 24 h under the conditions described in the “Cell culture” section, and viability was assessed by the MTT assay, as described in the “MTT method for cell viability assessment” section.

MCF-7 cell spheroids were maintained as described in the “Formation of MCF-7 cell spheroids” section. Next, spheroids were exposed for 30 or 60 min to culture medium only (no AlPc control) or to S444 at concentrations equivalent to 0.62, 1.25, or 2.50 μM AlPc. For each of these treatments, one-half of the wells containing spheroids was kept in the dark while the other half was irradiated with LED light (660 nm) at an energy density of 4.4 J/cm^2^. After the treatments, cell lysis was quantified by the amount of lactate dehydrogenase released in the culture supernatant as described in the “Lactate dehydrogenase method for cell lysis assessment” section.

### MTT method for cell viability assessment

Cell viability was assessed by the classical method of mitochondrial reduction of 3,4,5-dimethylthiazol-2,5 biphenyl tetrazolium bromide (MTT) by viable cells to an insoluble purple formazan [38]. Briefly, after receiving the respective treatment (see “Cell treatment design”), cells were washed twice with PBS and then incubated with 0.5 mg/mL MTT in culture medium for 2.5 h at 37 °C, 5 % CO_2_ and humidified atmosphere. Next, the MTT solution was removed, and formazan was extracted from cells with 200 μL of DMSO. The formazan-specific light absorption was then measured at 595 nm with a spectrophotometer (Spectramax M2, Molecular Devices, USA). This experiment was performed three times, and the results are expressed as a percentage relative to the control.

### Lactate dehydrogenase method for cell lysis assessment

The viability of spheroid cells was assessed by the quantification of lactate dehydrogenase activity by a colorimetric assay (CytoTox 96®, Promega Corp., Madison, WI, USA). Briefly, 40 μL of the supernatant of spheroid cultures were mixed with 40 μL of CytoTox 96®, and the mixture was kept in the dark for 30 min. Next, light absorption was recorded at 490 nm. The results were expressed as arbitrary units (au) of lactate dehydrogenase activity.

### Statistical analyses

All statistical analyses were performed with GraphPad Prism 5.0 software. Correlation between variables was analysed with the Pearson or Spearman test. Significant differences between groups were assessed by one-way analysis of variance (ANOVA) followed by Tukey or Bonferroni’s post-tests (*α* = 0.05). Results are expressed as mean ± standard error of the mean.

## Additional file

Additional file 1: Figure S1. Viability of non-cancerous (MCF-10A, human) and cancerous (MCF-7, human) cells exposed to different concentrations of S0 (nanoemulsion without AlPc) for 15 min, and then kept in the dark or irradiated (660 nm, 4.4 J/cm^2^). Cell viability was evaluated by the MTT method 24 h after treatment.
