# Supplementary figures and images for: Aluminium-phthalocyanine chloride nanoemulsions for anticancer photodynamic therapy: Development and in vitro activity against monolayers and spheroids of human mammary adenocarcinoma MCF-7 cells
Source: J Nanobiotechnology. 2015 May 13;13:36. doi: 10.1186/s12951-015-0095-3 (PMC4455699; doi:10.1186/s12951-015-0095-3)

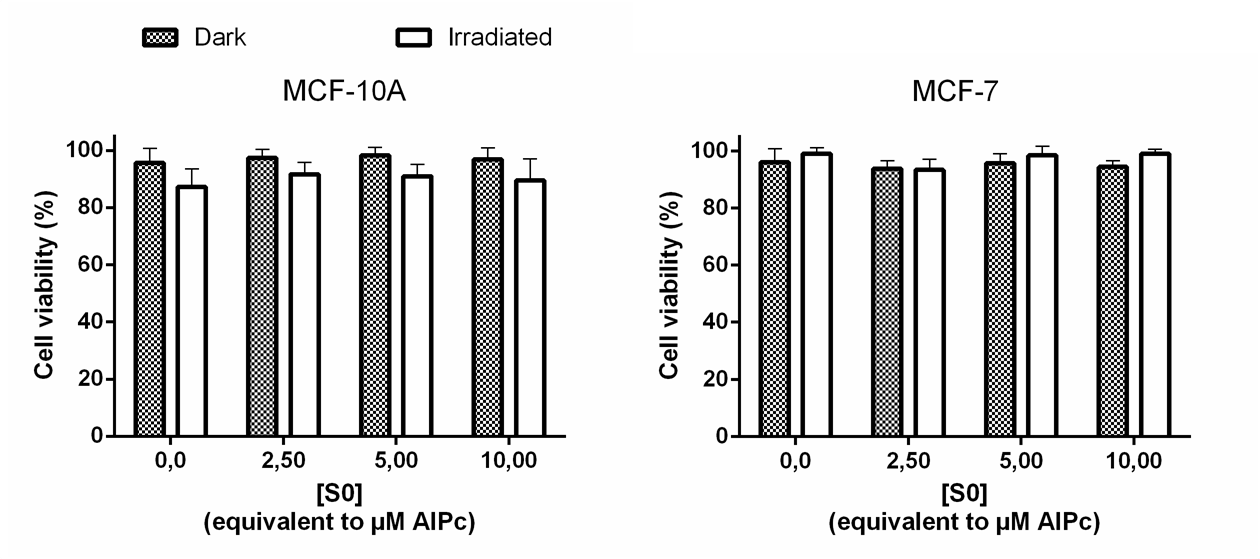

Supplement: Additional file 1: Figure S1. — Viability of non-cancerous (MCF-10A, human) and cancerous (MCF-7, human) cells exposed to different concentrations of S0 (nanoemulsion without AlPc) for 15 min, and then kept in the dark or irradiated (660 nm, 4.4 J/cm2). Cell viability was evaluated by the MTT method 24 h after treatment. [file 12951_2015_95_MOESM1_ESM.tiff]
